# Development of a community-based intervention for the control of Chagas disease based on peridomestic animal management: an eco-bio-social perspective

**DOI:** 10.1093/trstmh/tru202

**Published:** 2015-01-19

**Authors:** Sandra M. De Urioste-Stone, Pamela M. Pennington, Elizabeth Pellecer, Teresa M. Aguilar, Gabriela Samayoa, Hugo D. Perdomo, Hugo Enríquez, José G. Juárez

**Affiliations:** Centro de Estudios en Salud, Universidad del Valle de Guatemala, Guatemala 01015, Guatemala

**Keywords:** Cluster randomized, Participatory action research, PRECEDE-PROCEED, Rodent control, Vector control

## Abstract

**Background:**

Integrated vector management strategies depend on local eco-bio-social conditions, community participation, political will and inter-sectorial partnership. Previously identified risk factors for persistent *Triatoma dimidiata* infestation include the presence of rodents and chickens, tiled roofs, dirt floors, partial wall plastering and dog density.

**Methods:**

A community-based intervention was developed and implemented based on cyclical stakeholder and situational analyses. Intervention implementation and evaluation combined participatory action research and cluster randomized pre-test post-test experimental designs. The intervention included modified insecticide application, education regarding Chagas disease and risk factors, and participatory rodent control.

**Results:**

At final evaluation there was no significant difference in post-test triatomine infestation between intervention and control, keeping pre-test rodent and triatomine infestations constant. Knowledge levels regarding Chagas disease and prevention practices including rodent control, chicken management and health service access increased significantly only in intervention communities. The odds of nymph infection and rat infestation were 8.3 and 1.9-fold higher in control compared to intervention communities, respectively.

**Conclusion:**

Vector control measures without reservoir control are insufficient to reduce transmission risk in areas with persistent triatomine infestation. This integrated vector management program can complement house improvement initiatives by prioritizing households with risk factors such as tiled roofs. Requirement for active participation and multi-sectorial coordination poses implementation challenges.

## Introduction

Chagas disease transmission by triatomine bugs persists among the poorest populations in Latin America,^1,2^ with little progress achieved in treatments to reduce morbidity and premature mortality.^3,4^ Significant but uneven advances in vector control were accomplished through indoor residual spraying. However, sustainable surveillance and control interventions are needed in the long-term.^5,6^ It has been argued that public health interventions, as well as prevention/control efforts, should actively incorporate community participation, while considering knowledge and perceptions of populations at risk.^7^ Social, economic and political factors have increasingly been recognized as risk factors linked to the disease. However, research and control efforts focusing on these factors have remained scarce.^2,8–10^ Limited knowledge about the disease combined with poor housing conditions and deficient sanitary surroundings provide environments where triatomine bugs thrive and continue to pose challenges for sustainable control.^7^

In Guatemala, there are regions where *Triatoma dimidiata* infestations have remained above 20%, despite multiple insecticide treatments over the past decade.^11^ The eco-bio-social factors associated with persistent intra-domiciliary *T. dimidiata* infestation were determined in one such region of Guatemala.^12^ These factors included ownership of more than one dog, the presence of rodents, coffee trees, partially plastered walls, dirt floors and tiled roofs. In addition, dogs and rodents (*Mus musculus* and *Rattus rattus*) were found to be infected with *Trypanosoma cruzi*, and identified as blood meal sources for triatomines.^12^ Thus, these animals may play a role as parasite reservoirs in the household.

These previously identified risk factors for transmission guided the design of the intervention to address them through an integrated approach promoting active participation of community members to ensure sustainable Chagas disease control. We hypothesized that the risk of transmission could be minimized by providing access to tools and knowledge to reduce rodents as reservoirs. This work describes 1) eco-bio-social and institutional situational analyzes; 2) community-based participatory processes to develop, implement and evaluate the intervention; 3) changes in knowledge, attitudes and practices; 4) intervention effects on rodent infestations; 5) analysis of early instar infection; 6) reflections from participants and key stakeholders on intervention implementation.

## Materials and methods

### Situational analysis

In 2011, a knowledge, attitude and practices (KAP) survey was performed to identify eco-bio-social risk factors for *T. dimidiata* infestation in 472 households among 30 communities in the municipality of Comapa and two in the municipality of Zapotitlán, department of Jutiapa, in eastern Guatemala.^12^ A follow-up KAP and animal survey in a subset of 248 households was conducted to better understand the role of rodents and domestic animals.

Twelve face-to-face semi-structured interviews^13^ were performed with key stakeholders in Jutiapa and Guatemala City, between November 2010 and April 2011. Interviews generated information about policy, strategic actions, and collaborative efforts between stakeholders, as related to Chagas disease. Information enabled mapping the stakeholder environment and policy framework on Chagas disease prevention. Individual written informed consent was obtained from participants before surveys, interviews and participatory activities. Group written consent was obtained before group meetings. Consents were obtained to photograph and video record activities. The study was performed in accordance with the Guide for the Care and Use of Laboratory Animals of the National Institutes of Health.

### Pre-test post-test control group study design

Eighteen communities from Comapa (Jutiapa, Guatemala) with infestation levels >15% during the situational analysis survey^12^ were randomly assigned to nine control and nine intervention groups. Twenty-four households per community (one community had only 21 households), representing a cluster, were selected using a probability systematic sampling design.^12,14^ All households simultaneously received pre-test (2012) and post-test (2014) KAP, entomological and rodent surveys, as described before.^12^ Pre-test post-test surveys were designed to develop indices measuring changes in participants' knowledge about Chagas disease, triatomine risk perceptions, and domestic and peridomestic animal management practices associated with triatomine presence (Supplementary Table 1).^12^

### Intervention

An integrated vector management (IVM) intervention was aligned with health and development programs in the region to reduce transmission risks associated with persistent infestation. Intervention development included a cyclical process of requesting community members' input, and engaging them in critical reflection regarding appropriate/appealing practices, based on the situational analysis of current practices regarding previously identified risk factors. The intervention (Supplementary Figure 1 and Supplementary information) integrated the following: 1) education regarding the disease and risk factors associated with infestation; 2) a modified participatory spraying method including tiled roofs and all walls to eliminate triatomine infestation potentially associated with rodent nests; 3) participatory education and training in mechanical rodent control; 4) participatory education and training in organic waste management combined with productive household activities (i.e. horticulture and chicken rearing); 5) a participant-based reflective process on the intervention. Selected strategies considered participants' input. Groups participating were heterogeneous (comprising men, women, children and mixed adults). Several children joined their parents during participatory meetings in intervention communities. Control communities were offered the standard Ministry of Health (MoH) insecticide application method that excludes external walls and tiled roofs, with deltamethrin, 25 mg active ingredient/m^2^ (Bayer Environmental Science, Lyon Cedex 09, France).

### Triatomine infection determination

Gut tissue of first, second and third instar nymphs, as an indicator of recent transmission risk, was processed for DNA extraction and polymerase chain reaction for *T. cruzi* minicircle DNA as previously described.^12^

### Participatory action research (PAR)

Participatory action research was used to aid perception and behaviour changes through active stakeholder participation.^15^ Participants shared their knowledge, acquired new knowledge, recorded changes they practiced at home, and reflected upon the learning-change process. This approach was intended to build ownership of information and commitment to the learning-change-action effort.^15^

### Stakeholder intervention perceptions: in-depth interviews

Seven face-to-face, unstructured interviews were performed with local governmental and non-governmental representatives between December 2012 and April 2013 in Comapa, Jutiapa, to understand local context regarding institutional roles and experiences in development projects. Semi-structured interviews with key informants (n=19) and selected participants (n=29) were conducted from October 2013 to March 2014 in order to understand intervention implementation and adoption of new practices by participants. Participants were selected to represent all communities based on maximum variation^16^ considering gender, participation level, age and marital status.

### Data mining and analysis

Quantitative and qualitative databases were created in SPSS 20.0 (IBM, Armonk, New York, USA) and NVivo 9 (QSR International Pty Ltd. Doncaster, Victoria, Australia). Survey data were double checked before data entry. Codebooks were developed for each database. Indices to measure changes in KAP regarding triatomine prevention were created in SPSS 20.0 (IBM) based on combinations of questions from the KAP surveys performed in 2012 (pre-test) and 2014 (post-test) (Supplementary Table 1). Each index was analysed by Student's t test to determine differences between control (n=198) and intervention (n=194) communities at each time point. Domiciliary triatomine infestation was analysed by binary logistic regression with the following covariates: community (cluster), coverage (percentage of households participating per total household number in the community and insecticide application acceptance), domiciliary triatomine and rodent infestation at baseline. Odds ratios were calculated for nymph infection and rat infestations.

Interviews and intervention participatory group meetings were recorded and later transcribed verbatim. Field notes, transcriptions, matrices and reflective notes from intervention participatory group meetings were encoded into NVivo 9 (QSR Int.). Data analysis was performed simultaneously with data generation.^13^ Axial coding was used to generate categories and themes^17^ based on the Predisposing, Reinforcing, and Enabling Constructs in Educational/Environmental Diagnosis and Evaluation–Policy, Regulatory, and Organizational Constructs in Educational and Environmental Development (PRECEDE-PROCEED) model.^18^ Comparative matrices were elaborated to synthesize eco-bio-social data and advanced search routines were conducted for in-depth analysis.^19^

## Results

### Chagas disease control stakeholder context and policy analysis

**Figure 1.**
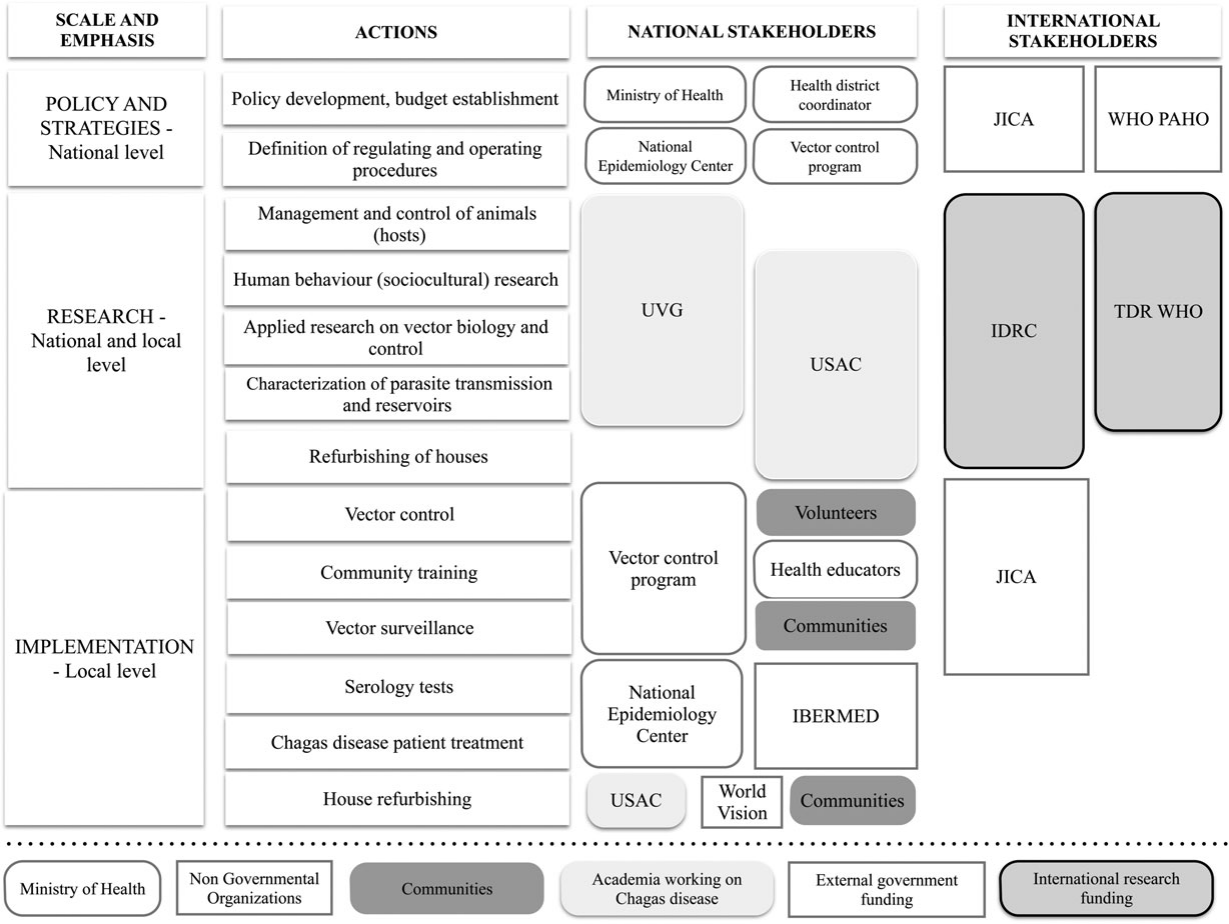
Local, national and international stakeholders with activities related to Chagas disease control from 2010–2011 in Comapa, Jutiapa, Guatemala. IBERMED: Médicos con Iberoamérica; IDRC: International Development Research Centre; JICA: Japanese International Cooperation Agency; PAHO: Pan American Health Organization; TDR: Special Programme for Research and Training in Tropical Diseases; USAC: Universidad de San Carlos de Guatemala; UVG: Universidad del Valle de Guatemala.

Figure 1 presents local (department of Jutiapa), national (Guatemala) and international stakeholders involved in Chagas disease control. This figure shows national policy development; local prevention, monitoring and disease treatment; research carried out locally and nationally. Disease prevention strategies included a revised version of the National Vector Control Guidelines and community-based triatomine surveillance. National Chagas Disease Control Program, the Japanese International Cooperative Agency (JICA) and World Vision (a non-governmental organization) have actively worked on control programs for Chagas disease in the past decade.

### Situational analysis KAPs

Limited knowledge regarding the disease and transmission was identified during the situational analysis. Only 240/472 (50.7%) participants mentioned some knowledge about the disease transmitted by triatomines or had heard about Chagas disease (Table 1). When prompted with additional questions regarding transmission, a total of 242 responded. Of these, 87 (36%) said they knew how the disease was transmitted and mentioned primarily health centre and vector personnel as sources of information (Table 1). When asked additional questions about transmission, a total of 96 responded. Of these, 75 (78.1%) participants mentioned vector-borne transmission; only 8 (8.3%) had the misconception of person-to-person transmission. In contrast, there was widespread awareness regarding triatomines. When shown triatomine pictures, 419/470 (89.1%) correctly recognized them (Table 1). Most (87.3%) responded they had heard about triatomines (Table 1). Of 412 who knew something about triatomines, 410 (99.5%) knew they posed a danger to their health and 311 (75.5%) mentioned they did something to protect themselves from triatomines. When asked what they would do to protect themselves from triatomines, frequent answers included spraying insecticides, plastering walls, killing bugs and taking the domestic animals outside the house (Table 1). Almost half of 472 participants reported the presence of chickens and dogs inside the house during the day (Table 1). Of these, 173 (36.7%) had chicken coops. Twelve percent (78/638) of all collected triatomines were found in chicken coops. Of 33 households with peridomestic infestations, 12 (36.4%) had infestations in coops with adobe, bajareque (mud and sticks), thatch or cinder block wall materials. Regarding rodents as risk factors, 57/304 participants (18.8%) did not know if mice or rats were hazardous (Table 2). Of 190 who knew rodents were hazardous, 128 (67.3%) indicated rodents carry diseases, 20 (10.5%) mentioned that the rodents carry fleas and 28 (14.7%) recognized rodents consume food destined for humans (Table 2). Regarding rodent control practices, both mice and rats were controlled with the same methods, mainly using poisons. Only 10/241 (4.1%) mentioned using traps to control rats. Cats were mentioned by one third of respondents as a rodent control method. Of 246 households surveyed for rodents, 118 (48.0%) and 52 (21.1%) had detectable *Mus musculus* and *Rattus rattus* infestations, respectively.

**Table 1.** Knowledge, attitudes and practices (KAP) regarding Chagas disease, triatomines and infestation risk factors (situational analysis)

Knowledge, attitudes and practices	Responses	No. positive responses/total (%)
Knowledge about Chagas disease	Know how Chagas disease is transmitted	87/242 (36.0)
	Know it is transmitted through contact with sick people	8/96 (8.3)
	Know it is transmitted by triatomines (‘chinche’)	75/96 (78.1)
	Know it is transmitted by blood transfusion	14/96 (14.6)
	Is acquainted with infected people	42/345 (12.2)
	Know people progressively get sick and die after infection	121/246 (49.2)
	Know people can get a fever after infection	32/246 (13.0)
	Know an eye can swell after infection	16/246 (6.5)
	Know people can feel malaise after infection	13/246 (5.3)
Knowledge and prevention practices related to triatomines	Can recognize triatomines when shown photos	419/470 (89.1)
	Has heard about triatomines (‘chinche picuda, talaje, telepate’)	411/471 (87.3)
	Know that triatomines pose a danger to health	410/412 (99.5)
	Know what triatomines feed on (animal blood)	212/412 (51.5)
	Know what triatomines feed on (human blood)	342/412 (83.0)
	Does something to protect self from triatomines	311/412 (75.5)
	Kill triatomines when found	331/412 (80.3)
	Capture triatomines and know where to take them when found	89/412 (21.6)
	Let triatomines go when found	3/412 (0.7)
	Go to the health centre when someone in the family is bitten by a triatomine	229/413 (55.4)
	Go to the hospital when someone in the family is bitten by a triatomine	17/413 (4.1)
	Believe Chagas disease transmission can be prevented by spraying insecticide	132/246 (53.7)^a^
	Believe Chagas disease transmission can be prevented by plastering walls	82/246 (33.3)^a^
	Believe Chagas disease transmission can be prevented by killing triatomines	29/246 (11.8)^a^
	Believe Chagas disease transmission can be prevented by taking the animals outside the house	29/246 (11.8)^a^
	Chickens come inside the house during the day	228/472 (48.3)
	Ducks come inside the house during the day	70/472 (14.8)
	Dogs come inside the house during the day	219/472 (46.4)
	Chickens come inside the house during the night	92/472 (19.5)
	Ducks come inside the house during the night	19/472 (4.0)
	Dogs come inside the house during the night	43/472 (9.1)

Questions with denominators <472 were dependent on a positive response to a previous question.

^a^ Some individuals reported more than one method to prevent Chagas disease transmission.

**Table 2.** Knowledge, attitudes and practices (KAP) regarding rodents as hazards and their control (situational analysis)

Knowledge, attitudes and practices	Responses	No. responses/total (%)
Knowledge regarding rodents as hazards^a^	Don't know if rodents are hazardous	57/304 (18.8)
	Rodents carry diseases	128/190 (67.3)
	Rodents carry fleas	20/190 (10.5)
	Rodents consume human foods	28/190 (14.7)
Practices regarding rodent control^b^	Uses traps to control rats	10/241 (4.1)
	Uses traps to control mice	10/246 (4.1)
	Does nothing to control rats	14/241 (5.8)
	Does nothing to control mice	15/246 (6.1)
	Uses poisons to control rats	153/246 (62.2)
	Uses poisons to control mice	165/253 (65.2)
	Uses cats for mouse control	86/249 (34.5)
	Uses cats for rat control	76/244 (31.1)
Practices regarding organic waste management^b^	Burns leftovers	2/248 (0.8)
	Buries leftovers	1/248 (0.4)
	Throws away leftovers	11/248 (4.4)
	Feeds leftovers to animals	143/248 (57.7)
	No leftovers	72/248 (29.0)
	Uses leftovers for composting	1/248 (0.4)

^a^ Baseline knowledge, attitudes and practices.

^b^ Follow-up knowledge, attitudes and practices.

### Intervention effects on triatomine infestation and infection

In 2012, domiciliary triatomine infestation was 19.3±10.2% and 19.0±9.0% in intervention (n=194) and control (n=199) communities, respectively. In 2014, infestation was 7.9±7.0% and 5.9±5.8% in intervention and control communities, respectively. Infestation was not significantly different between groups when controlling for baseline rodent and triatomine infestations, cluster and coverage (% households participating among the community and households accepting insecticide applications). Odds (95% CI) of finding bugs in a house belonging to the intervention was 10.6 times greater (3.2–34.8, 95% CI) if it was previously infested (Table 3). There were no significant odds of finding bugs in a control house if it was previously infested. Re-infestation in the intervention communities had 2.6 (1.4–4.9 95% CI) higher odds to occur in houses with tiled roofs (7/16), whereas in control communities they were not associated with this type of roof (1/12).

**Table 3.** Odds ratios of early instar infection with *T. cruzi* and rat infestations in control compared to intervention communities and triatomine re-infestation of previously infested households in the intervention

Outcome	OR	95% CI
Persistent household-level re-infestation (pre-infestation vs post-infestation)	10.6	3.2–34.8
Early instar infection (control vs intervention)	8.3	2.4–28.4
Rat infestation (control vs intervention)	1.9	1.09–3.45

Infection was detected by PCR in 4/37 (10.8%) 1^st^ to 3^rd^ instar nymphs from intervention households and 17/34 (50.0%) from control. There was 8.3-fold (2.4–28.4, 95% CI) higher odds of finding infected early instars in the control than the intervention (Table 3).

### Intervention effects on rodent infestation

In 2014, *R. rattus* infestation was 30/192 (15.0%) in control and 16/190 (8.4%) in intervention communities, with 1.9 (1.09–3.45, OR 95% CI) higher odds of finding rats in control than intervention communities. There was no difference in *Mus musculus* total infestation, with 76/192 (39.6%) in control and 63/190 (33.2%) in intervention communities. However, intervention communities had fewer households with more than two mice, with only 7/190 (3.7%), compared to 20/192 (10.4%) in the control communities.

### Changes in knowledge, attitudes and practices

**Figure 2.**
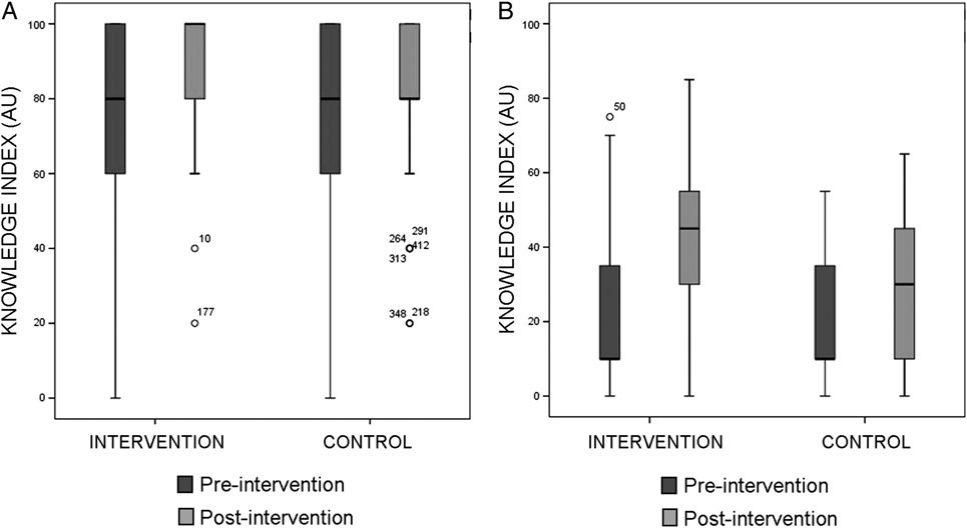
Indices on knowledge about triatomines and Chagas disease in intervention and control communities pre- and post-intervention. (A) Knowledge regarding triatomine biology. (B) Knowledge regarding Chagas disease. Boxplots show index scores median, interquartile range, minimum and maximum values. Score arbitrary units are defined in Supplementary Table 1. Numbered circles represent individual outliers.

At baseline in 2012 there was a significant difference between intervention and control communities in knowledge regarding triatomine biology (Student's t test, p<0.05) (Figure 2A). No significant difference between groups was detected regarding knowledge and attitudes towards the disease at baseline (Figure 2B; p>0.5). In the post-test survey in 2014, there was a significant difference between intervention and control groups in the knowledge regarding triatomine biology and Chagas disease (p<0.001).

**Figure 3.**
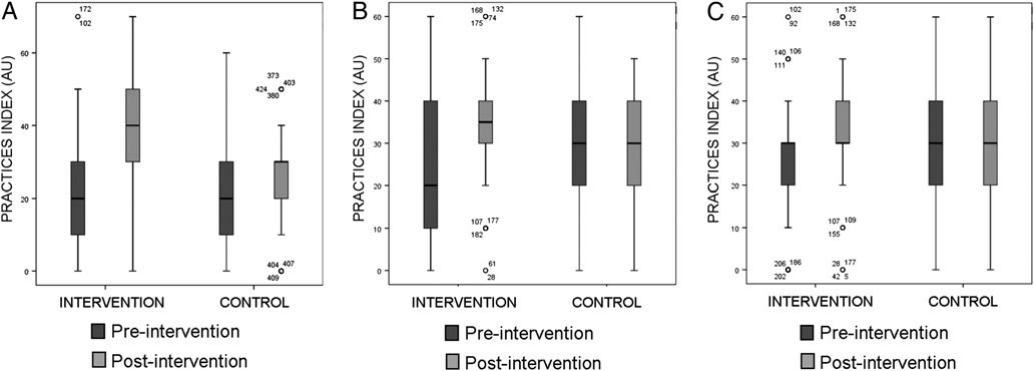
Indices measuring triatomine prevention practices related to (A) rodent control, (B) chicken management and (C) access to health care in intervention and control communities pre- and post-intervention. Boxplots show index scores median, interquartile range, minimum and maximum values. Score arbitrary units are defined in Supplementary Table 1. Numbered circles represent individual outliers.

At baseline, there was no significant difference between intervention and control groups in practices to protect themselves from the disease, which included rodent control measures (Figure 3A), chicken management (Figure 3B) or accessing health services (Figure 3C). In the post-test survey, there was a significant difference between intervention and control groups in prevention practices mentioned above (p<0.001). There was also a significant difference in knowledge regarding rodent biology (p<0.05) (data not shown).

In the final KAP survey, of 182 households in the intervention group who practiced rodent control, 142 (78%) mentioned using traps. Our environmental management recommendations were adopted by 51/67 (76%) households who had orchards, and 42/69 (60.9%) who produced compost. Of the 89 participants in the intervention who mentioned at the pre-test survey that rats carry diseases, 30 (33.7%) described using poisons and 73 (82%) described the use of traps, at the post-test survey. Of 92 participants in the control who mentioned rats carry diseases at the pre-test survey, 58 (63.0%) used poisons and 12 (13.0%) used traps, showing significantly different practices between groups at the post-test survey (Fisher's exact, p<0.001).

### Intervention sustainability and limitations

During group reflective sessions to enhance the PAR learning-reflection-action process (Table 4), participants identified intervention benefits, among which were: provision of traps for rodent control; spraying and cleaning for bug control; practices calendar; orientation on how to protect themselves from the triatomine bug. According to some participants, sustainability of the initiative will depend on collaboration between the health centre, local NGOs, other government agencies and community members, among others. Table 5 shows variable levels of participation as a percentage of meetings attended by a household member. The majority of households had a high participation level (96 of 216, 44%), followed by a medium participation level (93 of 216 households, 43%). Key barriers to participation included communication limitations and responsibilities towards caring for family members (children, older relation, husband). Sharing participation responsibilities across household members was demonstrated as a way to ensure participation at meetings.

**Table 4.** Tasks and timeline for household surveys, intervention implementation and intervention process evaluation

Task	Timeline
Pre-intervention KAP, entomological and rodent surveys	Sept–Nov 2012
Participatory meeting 1: project introduction, situational analysis and discussion of expectations	Oct 2012
Participatory meeting 2: strengths, weaknesses, opportunities, threats to understand the community context and stakeholder environment	Oct–Nov 2012
Insecticide application	Nov 2012–Feb 2013
Participatory meeting 3: education on Chagas disease symptoms, treatment and transmission routes	Nov–Dec 2012
Participatory meeting 4: education on biological and ecological aspects of triatomines and rodents, and their role as reservoirs	Feb–Apr 2013
House-to-house training on mechanical rodent control and delivery of rodent traps	Apr 2013
Participatory meeting 5: process reflection	May 2013
Participatory meeting 6: chicken management and education on composting	May–Jun 2013
Multi-sectoral guidelines to implement a family orchard	Jul–Aug 2013
Participatory meeting 7: final reflection	Aug–Oct 2013
Participant interviews	Oct 2013 and Mar 2014
Key stakeholder interviews	Oct 2013–Mar 2014
Post-intervention KAP, entomological and rodent surveys	Feb–Apr 2014

KAP: knowledge, attitudes and practices.

**Table 5.** Participation levels as a percentage of meetings attended by each household in the intervention program according to community

Community^a^	No. households/total (%) according to participation level		
	Low^b^	Medium^c^	High^d^
Santa Barbara	4/24 (16.7)	11/24 (45.8)	9/24 (37.5)
Copalapa	5/24 (20.8)	12/24 (50.0)	7/24 (29.2)
El Pinito	2/24 (8.3)	7/24 (29.2)	15/24 (62.5)
San Juan	0/24 (0.0)	15/24 (62.5)	9/24 (37.5)
Almolonga	5/24 (20.8)	6/24 (25.0)	13/24 (54.2)
El Tepenance	5/24 (20.8)	9/24 (37.5)	10/24 (41.7)
El Carrizo	2/24 (8.3)	12/24 (50.0)	10/24 (41.7)
Madre Cacao	0/24 (0.0)	12/24 (50.0)	12/24 (50.0)
San Antonio	4/24 (16.7)	9/24 (37.5)	11/24 (45.8)
Total	27/216 (12.5)	93/216 (43.1)	96/216 (44.4)

^a^ Community names correspond to household clusters (‘caserío’) within villages.

^b^ No. of households participating in 0-2 meetings.

^c^ No. of households participating in 3-5 meetings.

^d^ No. of households participating in 6-7 meetings.

## Discussion

Our study shows that development of multi-sectorial IVM strategies relevant to communities, combining insecticide application with education and rodent control, will reduce the transmission risk in the household. The community-based program shared knowledge between participants and researchers about risk factors, emphasizing the role of peridomestic animal management. The use of socio-culturally sensitive education approaches led to significant changes in knowledge and practices related to animal management. High adoption of rodent control methods and higher odds of rodent infestation in the control group suggest method effectiveness. Higher odds of early instar infection in control communities, despite similar triatomine infestations, suggest the combined strategies could reduce risk of infection to humans.

Community-based interventions have been evaluated over recent decades. In Paraguay, combining insecticide application and plastering reduced infestation with *Triatoma infestans*.^20^ In Mexico and Guatemala, interventions testing household ordering and plastering show variability in effectiveness due to adoption differences between and within communities.^21,22^ Even though housing improvement and education can reduce *T. dimidiata* vector habitats, peridomestic infestation foci persist as a threat for transmission.^22^ Rodent control could complement vector control programs by reducing peridomestic reservoirs, as proposed for *Triatoma brasiliensis*.^23^

The association of tiled roofs with households showing persistent infestation in intervention communities points to a potential role of rat nests among tiles stored nearby, as suggested in Costa Rica.^24^ It is possible that the modified insecticide application prevented triatomine dispersal from rodent nests found in the walls and focalized infestation to houses with nearby rodent nests associated with stored tiles. However, it cannot be determined if the modified spraying method alone could lead to this observed difference. Developing control strategies to target peridomestic re-infestation sources could ultimately increase control efficacy.^25^

The intervention appears to have reduced rat infestations and recent triatomine infections. Future blood meal analysis should clarify the effect of the intervention on triatomine feeding behavior and contact with humans as a proxy to transmission potential. To our knowledge, this is the first study implementing mechanical rodent control in the region. Given the high rodent infestation levels observed, there is a need to develop and implement community-based rodent control not only for Chagas disease control but also to promote healthy environments.^26^

Given the complexity of eco-bio-social determinants, control strategies should consider population well-being and incorporate participation of government and civil society.^27^ Furthermore, our approach requires the development of community-directed actions adapted to sociocultural and biophysical characteristics of the areas.^2,28^ Future implementation challenges will include 1) the need to involve multiple stakeholders, such as ministries and local NGOs; 2) resources such as insecticide and rodent traps; 3) active participation of community members with low literacy levels; 4) limited time and resources such as water.^29^

Our IVM strategy, if presented as a complete program with defined institutional roles, could increase the efficacy of the MoH control program by capitalizing on collaborations between MoH, community leaders and food security programs, with existing infrastructure and organization for the delivery of rodent control methods. The strategy could complement housing improvement and education efforts by focusing on households with identified persistent infestation risk factors, such as tiled roofs, and improving insecticide application methods to include all walls and peridomestic construction material storage.^21^ Future studies will determine if this strategy could result in a reduction of transmission risks not only for Chagas disease, but also for other diseases transmitted by rodents, such as leptospirosis and salmonellosis, contributing to community-wide well-being.

A limitation of this study is the short-term entomological evaluation. The study was designed to detect a decrease of infestation from 20% to 10% in control communities and from 20% to 5% in intervention communities 1.5 years after intervention completion.^30^ However, infestation levels were measured 7–9 months after intervention completion. Future entomological surveys will be needed to evaluate intervention long-term effects. Another potential limitation to detect differences could be the sharing of intervention fragments across control communities through ongoing food security, MoH programs and neighbours. The pre-test post-test survey results suggest these potential sources of contamination were not significant at this time, but should be considered in future evaluations.

### Conclusions

The community-based intervention promoted rodent and chicken management for the reduction of Chagas disease transmission in a region with persistent triatomine infestation. Main outcomes included: 1) increased knowledge regarding Chagas disease; 2) behavioural changes with adoption of practices for triatomine and rodent control; 3) reduced rat infestations; 4) focalized re-infestation to houses with tiled roofs; 5) reduced infection odds of early instars; 6) promotion of collaboration between communities, private business, government agencies, non-governmental organizations and universities. The results suggest transmission is reduced and provides evidence to the MoH to modify insecticide application methods and prioritize households with tiled roofs for housing improvement strategies. We recommend that participatory rodent control should be included as part of Chagas disease control strategies in areas with high rat infestations.

## Supplementary data

Supplementary data are available at Transactions Online (http://trstmh.oxfordjournals.org/).

Supplementary Data
