# Supplementary material for: Development of a community-based intervention for the control of Chagas disease based on peridomestic animal management: an eco-bio-social perspective
Source: Trans R Soc Trop Med Hyg. 2015 Jan 19;109(2):159–67. doi: 10.1093/trstmh/tru202 (PMC4299527; doi:10.1093/trstmh/tru202)
Supplement: Supplementary Data [file supp_109_2_159__index.html]

Supplementary Data 

# Development of a community-based intervention for the control of Chagas disease based on peridomestic animal management: an eco-bio-social perspective

## Supplementary Data

Supplementary Data

**Files in this Data Supplement:**

- Supplementary Data - Docx file
- Supplementary Figure 1 - tif file
- Supplementary Table 1 - docx file
