# Supplementary material for: Development of a community-based intervention for the control of Chagas disease based on peridomestic animal management: an eco-bio-social perspective
Source: Trans R Soc Trop Med Hyg. 2015 Jan 19;109(2):159–67. doi: 10.1093/trstmh/tru202 (PMC4299527; doi:10.1093/trstmh/tru202)
Supplement: Supplementary Data [file supp_tru202_tru202supp.docx]

**<H1>Supplementary information<H1>**

**<H1>Intervention methods<H1>**

**<H2>Quality assurance<H2>**

Triangulation using multiple and different sources to corroborate evidence^1^ was key to enhance credibility.^2^ We used the following approaches: 1) triangulation across multiple sources^3^ or by interviewing different stakeholders in the study area; 2) triangulation across methods by generating information through different research techniques (interviews, group discussions, survey, observation, biophysical measures); 3) analytical triangulation^4^ by including views and interpretations of the multidisciplinary team of researchers. Interviews were conducted until saturation or redundancy was achieved.^5^ Member checking^1,6^ was used by allowing participants to review emergent ideas during participatory group meetings and result sharing meetings. NVivo 9 (QSR International Pty Ltd. Doncaster, Victoria, Australia) database creation and maintenance helped enhance research transferability, dependability and confirmability.^6^

**<H2>Intervention description<H2>**

Following is a brief description of each intervention component.

**<H3> Education regarding Chagas disease and associated risk factors<H3>**

Participatory meetings used visual education material to respond to contextual literacy levels and learning styles. Meetings focused on Chagas disease transmission, symptoms and treatment, triatomine biology and the role of domestic and synanthropic animals in transmission. An animated video illustrated vector biology, including adult and immature stages, their habitats and blood feeding^7^. Risk factors discussed included dogs and chickens nesting inside the house.

**<H3> Participatory insecticide treatment<H3>**

Based on our analysis of vector control guidelines and the association of triatomines with rodents (mice and rats) and tiled roofs,^8^ we included spraying tiled roofs (potential rat nests) and internal and external walls (potential mouse and rat nests). All control group households received community-based insecticide spraying according to Ministry of Health (MoH) guidelines or *Triatoma dimidiata* control that do not include tiled roofs or external walls that are not protected by a roof. The intervention group received the same insecticide treatment as the control, except that all external walls were sprayed before spraying indoors, and all tiled roofs were sprayed after completing the internal wall spraying. As a public-private partnership, we coordinated a donation of K-Othrine (Bayer Environmental Science, Lyon Cedex 09, France) by Bayer, Hudson pumps (H.D. Hudson Manufacturing Company, Chicago, IL, USA) by the Japanese International Cooperative Agency and the availability of trained community volunteers and MoH vector control personnel to supervise spraying in both control and intervention groups. Spraying was performed in 525 households from 20 November 2012 to 1 February 2013. Thirteen (2.5%) of these households that were not accessible at that time were sprayed in May 2013 instead.

**<H3> Participatory mechanical rodent control program<H3>**

The rodent control program was collaboratively developed through discussions with study participants, zoonotic disease MoH personnel and rodent control experts. Use of chemicals was discounted due to the potential non-target adverse effects on cats and dogs, given that over one third of the participants report owning cats for rodent control (Table 2). The control program was implemented based on mechanical trapping and environmental management to reduce rodent habitats and food sources. The information included recommendations on grain storage, elevating and moving stored grain away from walls, removing all grain spilled below the metal silos and placing silos approximately 20 cm away from walls to prevent rodent nests, rodent-proofing food storage, and composting methods to promote reduction of leaf litter.

Each household was given a calendar to keep a record of daily activities they performed regarding rodent trapping and environmental management, using stickers with cartoons representing these activities (person cleaning, rodent trap, compost box and orchard). Meetings were conducted to discuss rodent ecology and biology as well as types of traps. Cages and two different sized snap traps (a small trap for mice and a larger one for rats) were selected to provide participants an option regarding the trapping method, given that different individuals voiced preferences in applying each method. Corn tortilla was recommended as bait, given that it is a household staple. Positioning of small snap traps and cage traps in corners on the floor and large snap traps in roof eaves was recommended to promote both mouse and rat control.

We visited each house for training on trap use, maintenance and cleaning, frequency of trap use and safety measures in handling the rodents once they were captured and sacrificed according to guidance on humane control of rodents^9^ of the Universities Federation of Animal Welfare. Participants were recommended to bury the carcasses with lime as a health promotion measure.

**<H3> Education and training regarding environmental management<H3>**

A more sustainable poultry production system was proposed to reduce chicken nests in the house, based on a modified chicken production model promoted by the Ministry of Agriculture and Range. This includes a chicken coop with composting to provide chickens with a protein source. Composting is used to manage organic waste and leaf litter to reduce rodent food and nesting sources. We recommended coops should not contain materials where triatomines could hide. Four sample chicken coops were built with chicken wire and zinc roofing in different communities. Household chicken coops were selected based on high participation levels and previous triatomine infestations. For those who could not leave the chickens outside their sleeping quarters, we recommended periodically checking chicken roosting sites for triatomines and to place them as far as possible from the beds.

The Guatemalan Ministry of Agriculture and Range and World Vision (a non-governmental organization) both had ongoing productivity and food security projects promoting family orchards and chicken production in the study region. As an incentive to promote composting, we coordinated with these institutions to give our participants the opportunity to receive technical training and seeds for orchard implementation. A multi-sectorial agreement was drafted and implemented with the Ministry of Agriculture and Range, and World Vision. In July 2012, study participants from intervention communities were invited to join the World Vision and Ministry of Agriculture and Range programs. Training included methods to prepare the soil and sow different seeds.

**<H1>References<H1>**

1. Creswell JW*.* Qualitative inquiry and research design: choosing among five approaches. Thousand Oaks, CA: Sage Publications, Inc; 2013. 472 p.

2. Flick U. Designing qualitative research. Thousand Oaks, CA: Sage Publications, Inc, 2007. 120 p.

3. Mertens DM. Research methods in education and psychology: Integrating diversity with quantitative, qualitative, and mixed methods. Thousand Oaks, CA: Sage Publications, Inc; 1998. 422 p.

4. Patton MQ. Qualitative research & evaluation methods. Thousand Oaks, CA: Sage Publications, Inc; 2002. 598 p.

5. Merriam SB. Qualitative research: A guide to design and implementation. San Francisco, CA: John Wiley & Sons, Inc; 2009.320 p.

6. Erlandson DA, Harris EL, Skipper BL et al. Doing naturalistic inquiry: A guide to methods. Newbury Park, CA: SAGE Publications, Inc; 1993. 198 p.

7. Ramsey Willoquet JM, Salgado Rodríguez L. Cada quién para su casa. La enfermedad de Chagas [in Spanish]. Morelos, Mexico: Instituto Nacional de Salud Pública; 2008. <http://www.hablamosdechagas.com.ar/galeria/cada-quien-para-su-casa-la-enfermedad-de-chagas/> [accessed 8 December 2014].

8. Bustamante DM, De Urioste-Stone SM, Juárez JG, Pennington PM. Ecological, social and biological risk factors for continued *Trypanosoma cruzi* transmission by *Triatoma dimidiata* in Guatemala. PLoS One 2014;9;e104599.

9. Universities Federation for Animal Welfare. Guiding principles in the humane control of rats and mice. 2008. http://www.ufaw.org.uk/rodents.php [accessed 3 December, 2014].

**Supplementary figure 1.** Images depicting the intervention components: (A) Chagas disease, triatomine and rodent control, biology education and participatory meetings; (B) participatory insecticide application; (C) mechanical rodent trapping; (D) orchard production; (E) and (F) chicken coop with compost system.
