# Supplementary figures and images for: Development of a community-based intervention for the control of Chagas disease based on peridomestic animal management: an eco-bio-social perspective
Source: Trans R Soc Trop Med Hyg. 2015 Jan 19;109(2):159–67. doi: 10.1093/trstmh/tru202 (PMC4299527; doi:10.1093/trstmh/tru202)

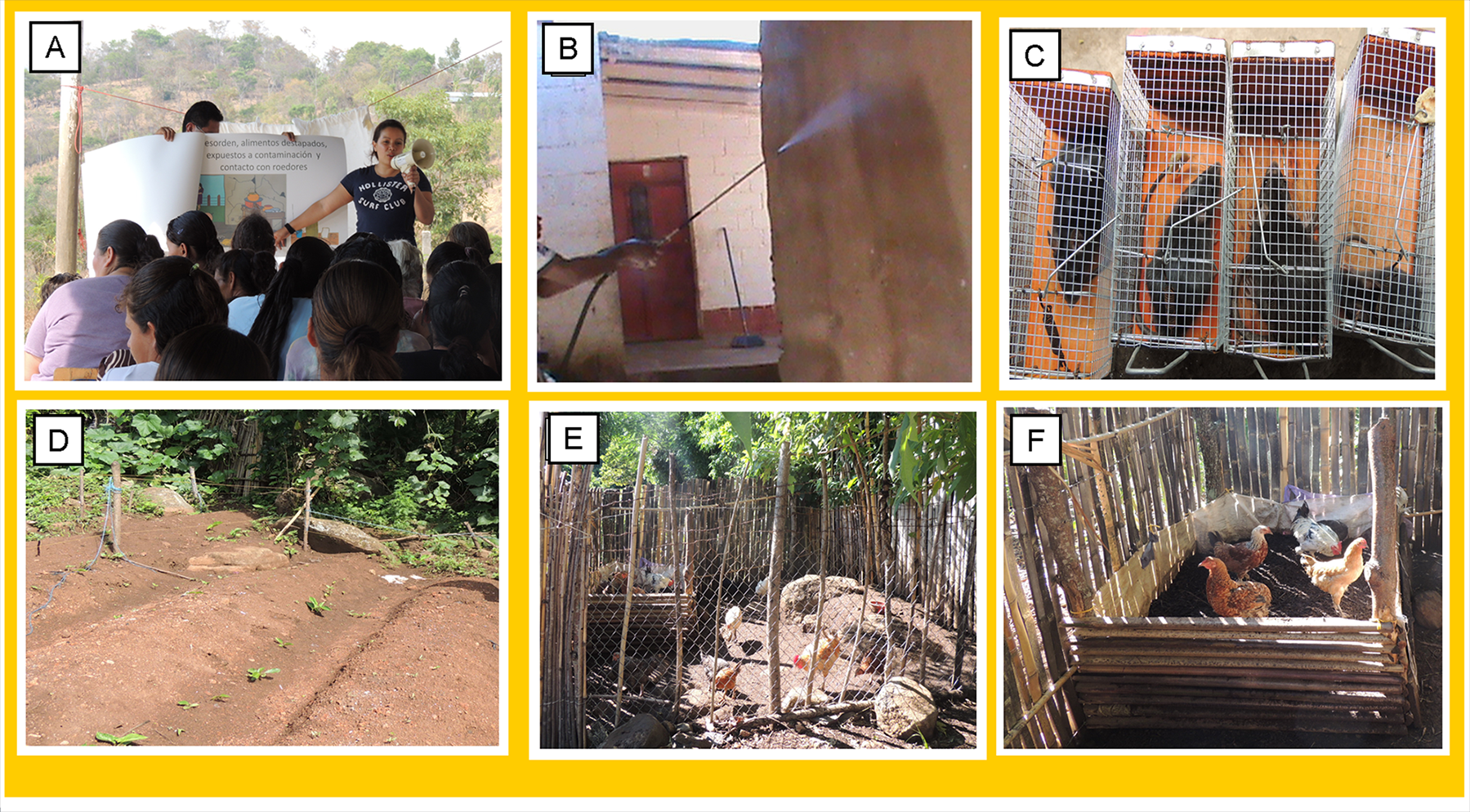

Supplement: Supplementary Data [file supp_tru202_tru202supp_fig1.tif]
