# Supplementary material for: Development of a community-based intervention for the control of Chagas disease based on peridomestic animal management: an eco-bio-social perspective
Source: Trans R Soc Trop Med Hyg. 2015 Jan 19;109(2):159–67. doi: 10.1093/trstmh/tru202 (PMC4299527; doi:10.1093/trstmh/tru202)
Supplement: Supplementary Data [file supp_tru202_tru202supp_table1.docx]

**Table 1.** Score composition of knowledge, attitude and practices (KAP) indices

| Index (scale)^b^ | Variable | Score^a^ |
| --- | --- | --- |
| Knowledge of Chagas disease  (0-120)  Knowledge regarding triatomines (0-100) | People get infected through triatomine contact | 15 |
|  | People get infected through blood transfusion | 15 |
|  | Infected people become sick until death | 10 |
|  | People can have a swollen eye after transmission | 10 |
|  | People can get a fever after transmission | 10 |
|  | People can suffer malaise after transmission | 10 |
|  | People can develop an enlarged heart after transmission | 10 |
|  | Awareness of triatomine bite due to lethargy | 10 |
|  | Awareness of triatomine bite due to itchiness | 10 |
|  | Awareness of triatomine bite due to local swelling | 10 |
|  | Knows something or has heard something about the disease transmitted by triatomines  Total score  Has seen triatomines  Has heard about triatomines  Knows triatomines are dangerous for health  Knows triatomines feed on human blood  Knows triatomines feed on the blood of other animals  Total score | 10  __  120  20  20  20  20  20  100 |
| Prevention practices with rodent control  (0-90) | Does something to protect themselves from triatomines | 10 |
|  | Sprays insecticides to protect themselves from triatomines | 10 |
|  | Improves walls to protect themselves from triatomines | 10 |
|  | Cleans to protect themselves from triatomines | 10 |
|  | Checks for triatomines to protect themselves | 10 |
|  | Kills triatomines to protect themselves | 10 |
|  | If they find a triatomine they kill it | 10 |
|  | Sets traps to prevent rats from entering the house | 10 |
|  | Sets traps to prevent mice from entering the house  Total score | 10  90 |
| Prevention practices with chicken management  (0-80) | Does something to protect themselves from triatomines | 10 |
|  | Sprays insecticides to protect themselves from triatomines | 10 |
|  | Improves walls to protect themselves from triatomines | 10 |
|  | Cleans to protect themselves from triatomines | 10 |
|  | Checks for triatomines to protect themselves | 10 |
|  | Kills triatomines to protect themselves | 10 |
|  | If they find a triatomine they kill it | 10 |
|  | Chickens do not come inside at night  Total score | 10  80 |
| Prevention practices with access to health services  (0-90) | Does something to protect themselves from triatomines | 10 |
|  | Sprays insecticides to protect themselves from triatomines | 10 |
|  | Improves walls to protect themselves from triatomines | 10 |
|  | Cleans to protect themselves from triatomines | 10 |
|  | Checks for triatomines to protect themselves | 10 |
|  | Kills triatomines to protect themselves | 10 |
|  | If they find a triatomine they kill it | 10 |
|  | If someone in the family is bitten by a triatomine they take them to the health centre | 10 |
|  | If someone in the family is bitten by a triatomine they take them to the hospital  Total score | 10  __  90 |

^a^ The score represents an arbitrary value assigned to each variable when present. The variable is scored as zero when absent.

^b^ Index scores are composed by the sum of all variables.
